# Gender-Specific Association between Tobacco Smoking and Central Obesity among 0.5 Million Chinese People: The China Kadoorie Biobank Study

**DOI:** 10.1371/journal.pone.0124586

**Published:** 2015-04-21

**Authors:** Jun Lv, Wei Chen, Dianjianyi Sun, Shengxu Li, Iona Y. Millwood, Margaret Smith, Yu Guo, Zheng Bian, Canqing Yu, Huiyan Zhou, Yunlong Tan, Junshi Chen, Zhengming Chen, Liming Li, China Kadoorie Biobank collaborative group

**Affiliations:** 1 Department of Epidemiology and Biostatistics, School of Public Health, Peking University Health Science Center, Beijing, China; 2 Department of Epidemiology, School of Public Health and Tropical Medicine, Tulane University, New Orleans, Louisiana, United States of America; 3 Clinical Trial Service Unit & Epidemiological Studies Unit (CTSU), Nuffield Department of Population Health, University of Oxford, United Kingdom; 4 Chinese Academy of Medical Sciences, Beijing, China; 5 China National Center for Food Safety Risk Assessment, Beijing, China; Hunter College, UNITED STATES

## Abstract

**Objectives:**

Lifestyle factors are well-known important modifiable risk factors for obesity; the association between tobacco smoking and central obesity, however, is largely unknown in the Chinese population. This study examined the relationship between smoking and central obesity in 0.5 million Chinese adults, a population with a low prevalence of general obesity, but a high prevalence of central obesity.

**Subjects:**

A total of 487,527 adults (200,564 males and 286,963 females), aged 30-79 years, were enrolled in the baseline survey of the China Kadoorie Biobank (CKB) Study conducted during 2004-2008. Waist circumference (WC) and WC/height ratio (WHtR) were used as measures of central obesity.

**Results:**

The prevalence of regular smokers was significantly higher among males (60.6%) than among females (2.2%). The prevalence of central obesity increased with age and BMI levels, with a significant gender difference (females>males). Of note, almost all obese adults (99.4%) were centrally obese regardless of gender. In multivariable regression analyses, adjusting for age, education, physical activity, alcohol use and survey site, regular smoking was inversely associated with BMI in males (standardized regression coefficients, β= -0.093, p<0.001) and females (β= -0.025, p<0.001). Of interest, in the BMI stratification analyses in 18 groups, all βs of regular smoking for WHtR were positive in both genders; the βs showed a significantly greater increasing trend with increasing BMI in males than in females. In the analyses with model adjustment for BMI, the positive associations between regular smoking and WHtR were stronger in males (β= 0.021, p<0.001) than in females (β= 0.008, p<0.001) (p<0.001 for gender difference). WC showed considerably consistent results.

**Conclusions:**

The data indicate that tobacco smoking is an important risk factor for central obesity, but the association is gender-specific and depends on the adjustment for general obesity.

## Introduction

Obesity has reached an epidemic level worldwide in both children and adult populations despite efforts of research and prevention for decades [1–5]. Obesity has gained importance because of its association with subsequent cardiovascular disease and all-cause mortality [6–8]. Obesity has also been shown to be associated with a number of cardiovascular disease risk factors, such as hypertension, dyslipidemia, type 2 diabetes, and insulin resistance, all known as metabolic syndrome components [9–12]. Asian populations have a relatively lower BMI compared with Western populations, but are predisposed to central or abdominal obesity [7,11–16]. This central fat accumulation is reported to be more strongly associated with cardiovascular disease risk factors and metabolic syndrome than peripheral fat distribution [11,12].

The rapid economic growth has been accompanied by urbanization, westernization of lifestyle behaviors and rising rates of obesity in China during the past couple of decades [4,5,17,18]. Based on secular trends in the prevalence of obesity among Chinese adults during 1993–2009, it seems that the obesity epidemic has not plateaued in China [5]. Central obesity and related cardiometabolic abnormalities have been considered an expression of a "Civilization Syndrome" [19]. The modern, urbanized society-related features, including unhealthy dietary patterns, lower physical activity, alcohol consumption and tobacco smoking, are well-known important modifiable lifestyle risk factors for obesity [1,17–21]. Despite a huge body of literature on the inverse association between smoking and general obesity [22–24], data are limited regarding the association between tobacco smoking and central obesity in Asians; no previous studies have focused on the smoking-central obesity association among adults in the Chinese population, with a low prevalence of general obesity, but a high prevalence of central obesity. The objective of the present study is to examine the relationship between tobacco smoking and central obesity measures in 0.5 million male and female adults enrolled in the China Kadoorie Biobank (CKB) Study.

## Methods

### Study Design and Participants

The CKB Study is a prospective study of chronic disease in China. Details of the CKB study design and characteristics of the study participants have been described elsewhere [25,26]. Briefly, 512,891 participants without major disabilities living in administrative units (rural villages or urban residential committees), aged 30–79 years (mean age: 51.5 years), were recruited in the baseline survey between 2004 and 2008 from five urban and five rural areas in China. Selection of the survey sites was based on local patterns of disease and exposure to certain risk factors, population stability, quality of death and disease registries, local commitment and capacity.

The current analysis included participants who had a weight between 30 kg and 160 kg, a height of 145–200 cm (males) and 140–200 cm (females), a BMI between 18.5 and 45.0, and a waist circumference between 50 cm and 150 cm. People who were very underweight or extremely obese or were at the extremes of the height distribution may have underlying metabolic or growth disorders. A total of 25,364 participants were excluded based on the above selection criteria, and 487,527 adults (200,564 males and 286,963 females) formed the sample for the current analysis.

Ethical approval for the CKB study was obtained from the Ethical Review Committee of the Chinese Center for Disease Control and Prevention (Beijing, China) and the Oxford Tropical Research Ethics Committee, University of Oxford (UK). In addition, approvals were obtained from the institutional research boards at the local Center for Disease Control and Prevention in each of the ten survey sites. Finally, written informed consent was obtained from all participants.

### Questionnaire survey

In the baseline survey, trained interviewers administered a standardized questionnaire using a laptop-based direct data-entry system, with built-in functions to avoid logical errors and missing items. The questionnaire included detailed questions on socio-demographic status, medical history and health status, smoking, alcohol consumption, physical activity, and other lifestyle behaviors.

Education was classified into five levels: Illiterate, elementary, middle school, high school, college and above. Total physical activity was converted into metabolic equivalent hours per day (MET-hours/day) spent on work, transportation, housework, non-sedentary recreation as described in our previous study [27]. For assessment of alcohol consumption, participants were asked how often they had drunk alcohol during the previous 12 months, and those who had not drunk weekly were asked if there was a period of at least a year prior to that when they had drunk some alcohol at least once a week. Participants were classified into four main drinking categories: 1) nondrinkers; 2) ex-drinkers; 3) occasional drinkers; and 4) weekly drinkers.

For assessment of tobacco consumption, participants were asked how often they had smoked tobacco at the time of the survey, and those who did not smoke or only smoked occasionally were asked if there was a period prior to that when they had smoked some tobacco on most days or daily. In the present analysis, participants were classified into four main smoking categories: 1) nonsmokers were defined as those who did not smoke currently and had not smoked more than 100 cigarettes during his/her lifetime; 2) ex-smokers as those who currently do not smoke or only smoked occasionally but had smoked on most days or daily in the past; 3) occasional smokers as those who do not smoke currently but had smoked occasionally in the past or had smoked at least 100 cigarettes during his/her lifetime, or those who currently smoke occasionally and had not smoked on most days or daily in the past; 4) regular smokers as those who currently smoke daily or on most days. Information on duration (years) of smoking and number of packs smoked per day was obtained for regular smokers, and pack-years was calculated as a measure of cumulative burden of smoking.

### Anthropometric measurements

Standing height and body weight were measured in light indoor clothing without shoes to the nearest 0.1 cm and 0.1 kg, respectively. Waist circumference (WC) was measured midway between the iliac crest and the lower rib margin at the end of normal expiration using a plastic flexible tape to the nearest 0.1 cm. All measurements were made by trained staff using a standard protocol and instruments. WC/height ratio (WHtR) was calculated as WC divided by height and was used as a measure of central obesity [12]. Body mass index (BMI) was calculated as weight in kilograms divided by the square of height in meters and was used as a measure of general obesity. Normal-weight (BMI = 18.5–23.9), over weight (BMI = 24.0–27.9) and obesity (BMI = 28.0–45.0) groups were defined based on recommendations of the Working Group on Obesity in China [7]. Central obesity was defined as WHtR> = 0.5 [11,12].

### Statistical analyses

Statistical analyses were performed using Stata Statistical Software (version 13.1, 2013, StataCorp LP., College Station, TX). All analyses were performed separately by gender groups. Descriptive data were presented as mean and standard deviation (SD) for continuous variables, and percentages for categorical variables. General linear models were used to test differences in continuous study variables between gender groups, adjusting for age, and survey sites for the descriptive data. Chi-square test was used to test the differences in categorical study variables between gender groups.

Multivariable linear regression analyses were performed (dependent variables = BMI, WC or WHtR; predictor = smoking) by gender, adjusting for age, survey site (included in the regression models as nine dummy variables using Qingdao as a reference), education (categorized as no formal school, elementary school, middle school, high school, or college and above), total physical activity (MET-hours/day) and alcohol use (nondrinker, ex-drinker, occasional drinker and weekly drinker). Smoking was included in the model as both a categorical variable (nonsmoking, ex-smoking, occasional smoking and regular smoking) and a continuous variable (pack-years). In order to assess the influence of adjustment for BMI on the association of tobacco smoking with WC and WHtR, males and females were stratified into 18 subgroups according to levels of BMI with equal numbers of subjects in each subgroup. Multivariable linear regression analyses were performed within each subgroup, adjusting for the above mentioned covariates. Standardized regression coefficients of regular smoking for WC and WHtR in the 18 BMI subgroups were plotted in males vs females. Linear regression models were used to assess significance of trends in the effect size of regular smoking on WC and WHtR across these 18 BMI subgroups; interaction regression models were used to test the difference in slopes of the regression lines between gender groups.

In sensitivity analyses, the association of tobacco smoking with WC and WHtR was examined using multivariable linear regression models, with and without BMI adjustment, by groups of normal-weight (BMI = 18.5–23.9), over-weight (BMI = 24.0–27.9), and obesity (BMI = 28.0–45.0), adjusting for covariates as mentioned above.

## Results

Table 1 summarizes the characteristics of the study cohort by gender. Males were older and had higher education than females (p<0.001); males had significantly higher levels of physical activity measured as MET-hours/day and prevalence of weekly alcohol drinkers than females. The prevalence of regular smokers was 27.5 times higher among males than that among females (60.6% vs 2.2%). Females had longer duration of smoking but lower packs/day and pack-years than males. Height, weight, and WC showed significant gender differences in mean values (males>females); however, BMI, WHtR, and the prevalence of central obesity were significantly lower among males than among females. Further, the prevalence of central obesity increased in the order of subgroups of normal-weight (BMI = 18.5–23.9), over-weight (BMI = 24.0–27.9), and obesity (BMI = 28.0–45.0) in both males and females, with significant gender differences in the normal-weight and over-weight groups (females>males). Of note, almost all Chinese adults (99.4%) with obesity in this cohort were centrally obese regardless of gender.

**Table 1:** Characteristics (mean±SD or %) of study variables by gender groups.

	Males (n = 200,564)	Females (n = 286,963)	*P* for gender difference
**Age (year)**	52.1 ± 10.8	50.7 ± 10.3	<0.001
**Education (%)**			<0.001
Illiterate and elementary	41.3	55.9	
Middle school and above	58.7	44.1	
**Physical activity (MET-hours/day)** ^a^	22.2 ± 15.3	20.5 ± 12.8	<0.001
**Alcohol use (%)**			<0.001
Nondrinker	19.7	63.0	
Ex-regular drinker	8.7	0.9	
Occasional drinker	38.0	34.1	
Regular drinker	33.7	2.0	
**Tobacco use**			<0.001
Nonsmoker	14.5	95.2	
Ex-regular smoker	13.4	0.8	
Occasional smoker	11.4	1.8	
Regular smoker	60.6	2.2	
**Regular smoker**			
Duration of smoking (years)	28.4 ± 10.9	29.6 ± 15.5	<0.001
Packs/day	0.9 ± 0.5	0.5 ± 0.4	<0.001
Pack-years/1000	9.5 ± 6.9	5.5 ± 5.4	<0.001
**Height (cm)** ^a^	165.3 ± 6.4	154.4 ± 5.7	<0.001
**Weight (kg)** ^a^	65.0 ± 10.4	57.5 ± 8.9	<0.001
**BMI (kg/m** ^**2**^ **)** ^a^	23.7 ± 3.1	24.1 ± 3.2	<0.001
**Waist circumference (cm)** ^a^	82.7 ± 9.4	79.8 ± 9.1	<0.001
**WHtR ratio** ^a^	0.500 ± 0.054	0.517 ± 0.060	<0.001
**Central obesity (%)** ^b^	48.8	58.2	<0.001
**Central obesity (%)** ^b^			
BMI 18.5–23.9	19.1	30.3	<0.001
BMI 24.0–27.9	84.0	85.8	<0.001
BMI 28.0–45.0	99.4	99.4	0.878

^a^: *P*-values for gender difference were adjusted for age and survey site;

^b^: Central obesity was defined as WHtR≥0.5.

MET-hours/day: metabolic equivalent hours per day

WHtR: waist circumference/height ratio

**Fig 1:**
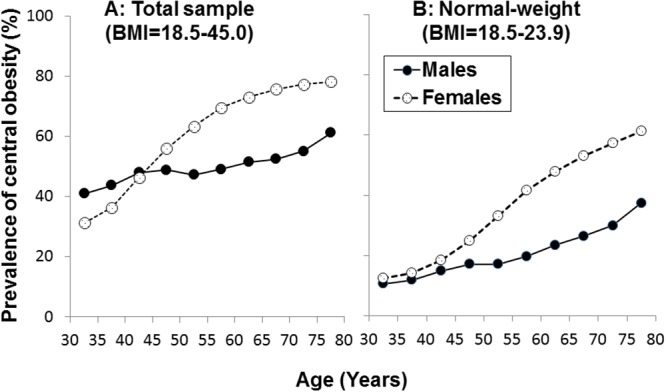
Prevalence of central obesity in the total sample (BMI = 18.5–45.0) (A) and normal-weight adults (BMI = 18.5–23.9) (B).

Fig 1. presents the prevalence of central obesity by gender and age in the total sample (A) and among individuals with normal-weight (B). In the total sample, the prevalence of central obesity increased with age, and females showed a significantly higher prevalence than males at ages above 45 years. Among normal-weight individuals, the prevalence of central obesity increased with age and was significantly higher among females than among males in all age groups, with a steeper increase in females than in males.

Table 2 shows mean levels of BMI, WC and WhtR by gender and smoking groups. Compared to nonsmokers, regular smokers had significantly lower values of BMI in males and females; the differences in WC and WHtR, however, showed an opposite direction in meals (regular smokers<nonsmokers) and females (regular smokers>nonsmokers). It has to be noted that the difference in unadjusted BMI between nonsmokers and regular smokers was greater in males (24.13–23.4 = 0.73) than in females (24.10–23.9 = 0.20). Apparently, the lower BMI resulted, at least partly, in lower WC and WHtR in male regular smokers.

**Table 2:** Unadjusted means±SD of adiposity measures by gender and smoking groups.

	Males			Females		
	BMI(kg/m^2^)	WC(cm)	WHtR	BMI(kg/m^2^)	WC(cm)	WHtR
Nonsmoker	24.13±3.0	83.8±9.3	0.507 ± 0.053	24.10±3.2	79.7±9.1	0.516 ± 0.060
Ex-smoker	24.4±3.1	85.0±9.7	0.515 ± 0.056	24.9±3.5	83.6±9.8	0.552 ± 0.063
Occasional smoker	24.1±3.0	83.4±9.0	0.505 ± 0.052	24.3±3.4	80.3±9.3	0.524 ± 0.061
Regular smoker	23.4±3.0	81.9±9.3	0.495 ± 0.053	23.9±3.4	80.4±9.6	0.528 ± 0.061
P-value^a^	<0.001	<0.001	<0.001	<0.001	<0.001	<0.001

WC: waist circumference

WHtR: waist circumference/height ratio

^a^: difference between nonsmokers and regular smokers, adjusted for age and survey sites.

Gender differences in means were all significant (P<0.001), adjusted for age and survey sites.

Table 3 presents linear regression coefficients of smoking for BMI, WC and WHtR by gender groups, adjusting for age, education, physical activity, alcohol use and survey site. Compared with non-smoking, ex-smoking was positively associated with BMI, WC and WHtR in males and females (p<0.001), but regular smoking was inversely associated with BMI, WC and WHtR in both gender groups (p<0.001). Occasional smoking was positively associated with all adiposity measures in males and females except for BMI in males. Pack-yeas was inversely associated with BMI, WC and WHtR in both gender groups (p<0.001) except for WC in males (positively). Gender differences in standardized regression coefficients of ex-smoking and regular smoking for WC and WHtR (males>females) were all significant; gender differences in standardized regression coefficients of pack-years were significant for BMI (males>females) and WC (an opposite direction). The regression coefficients of pack-years vs regular smoking for WC had different signs (0.006 vs -0.044) among males. The explanation is that the associations of light and heavy regular smoking and the duration of smoking with BMI may be different, and the large sample is highly sensitive to detect the change in the effect. Another explanation is that body height might have an influence in this regard when compared with the regression coefficient for WhtR (-0.007*, p = 0.011) in males.

**Table 3:** Standardized (upper) and unstandardized (lower) linear regression coefficients (95% confidence interval) for BMI, WC and WHtR by gender groups.

Independent variable	BMI		WC^a^		WHtR^a^	
	Males	Females	Males	Females	Males	Females
**Model 1**						
Non-smoking (reference)	—	—	—	—	—	—
Ex-smoking	0.037**	0.009** ^†^	0.057**	0.015** ^†^	0.049**	0.014** ^†^
	0.328**	0.323**	1.567**	1.488**	0.008**	0.009**
	(0.280, 0.377)	(0.195, 0.451)	(1.418, 1.715)	(1.135, 1.840)	(0.007, 0.009)	(0.007, 0.012)
Occasional smoking	-0.001	0.006**	0.006*	0.009**	0.007*	0.009**
	-0.006	0.144**	0.181*	0.612**	0.001*	0.004**
	(-0.056, 0.045)	(0.056, 0.233)	(0.027, 0.334)	(0.370, 0.854)	(0.0002, 0.002)	(0.003, 0.006)
Regular smoking	-0.093**	-0.025** ^†^	-0.044**	-0.010** ^†^	-0.061**	-0.011** ^†^
	-0.582**	-0.548**	-0.853**	-0.626**	-0.007**	-0.005**
	(-0.620, -0.544)	(-0.629, -0.467)	(-0.970, -0.736)	(-0.849, -0.403)	(-0.007, -0.006)	(-0.006, -0.003)
**Model 2** ^b^						
Pack-years/1000	-0.031**	-0.022** ^†^	0.006*	-0.008** ^†^	-0.007*	-0.010**
	-0.013**	-0.062**	0.007*	-0.066**	-0.00005*	-0.001**
	(-0.015, -0.011)	(-0.073, -0.052)	(0.001, 0.014)	(-0.095, -0.038)	(-0.00009, -0.00001)	(-0.001, -0.0003)

WC: waist circumference

WHtR: waist circumference/height ratio

* p<0.05,

** p<0.01 for coefficients different from 0

^†^ P<0.05 for gender difference

^a^: BMI was not included in the models for adjustment.

^b^: Only nonsmokers and regular smokers were included.

Age, education, physical activity, alcohol use and survey site were included in models for adjustment.

**Fig 2:**
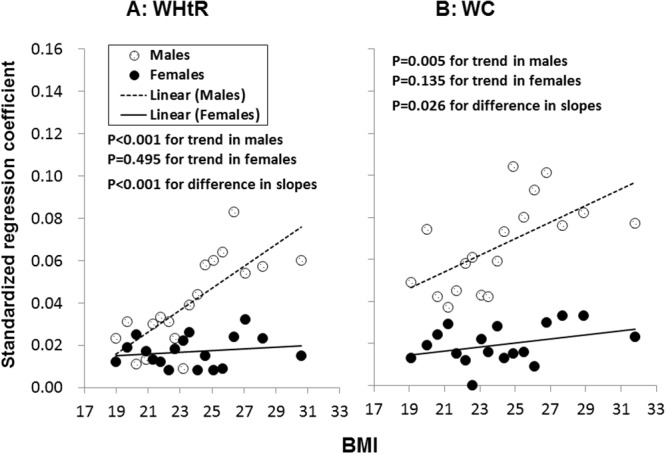
Standardized regression coefficients of regular smokers for WHtR (A) and WC (B) by 18 BMI groups in males and females: Standardized regression coefficients greater than 0.026 for male smokers and 0.015 for female smokers were significant (p<0.05).

In order to assess the influence of adjustment for BMI on the association analyses of smoking with WC and WHtR, males and females were stratified into 18 subgroups with equal numbers of subjects in each subgroup according to levels of BMI. Fig 2. depicts standardized regression coefficients of regular smoking for WHtR (A) and WC (B) by 18 BMI groups among males and females, adjusted for age, education, physical activity, alcohol use and survey site. The number of adults was 11,142–11,143 in the male subgroups and 15,942–15,943 in the female subgroups. The standardized regression coefficients of regular smoking were all positive in males and females and showed a significant increasing trend with increasing BMI for WHtR and WC in males, but not in females; the slopes of the regression lines were significantly different between males and females. It should be noted that the association parameters in the BMI stratification analysis, a powerful and reliable adjustment method, were completely different (opposite) from those in the regression analyses for WC and WHtR in Table 3 without BMI adjustment in the model.

In order to confirm the results of BMI stratification analyses, a model adjustment for BMI was used. Table 4 shows regression coefficients of smoking for WC and WHtR. When BMI was included in the regression models, both regular smoking and pack-years showed positive associations with WC and WHtR in males and females. The differences in standardized regression coefficients of regular smoking and pack-years between males and females in Table 4 were all significant for both WC and WHtR (males>females, p<0.01). The results of the model adjustment for BMI with respect to regression coefficients of regular smoking were substantially consistent with those of the BMI stratification analyses shown in Fig 2.

**Table 4:** Standardized (upper) and unstandardized (lower) linear regression coefficients (95% confidence interval) for WC and WHtR by gender groups, with adjustment for BMI.

Independent variable	WC		WHtR	
	Males	Females	Males	Females
**Model 1**				
Nonsmoking	—	—	—	—
Ex-smoking	0.026**	0.008** ^†^	0.017**	0.007** ^†^
	0.717**	0.753**	0.003**	0.005**
	(0.639, 0.795)	(0.555, 0.952)	(0.002, 0.003)	(0.003, 0.006)
Occasional smoking	0.007**	0.004**	0.007**	0.005**
	0.195**	0.284**	0.001**	0.002**
	(0.114, 0.276)	(0.147, 0.420)	(0.001, 0.002)	(0.001, 0.003)
Regular smoking	0.034**	0.010** ^†^	0.020**	0.008** ^†^
	0.654**	0.619**	0.002**	0.003**
	(0.593, 0.716)	(0.494, 0.745)	(0.002, 0.003)	(0.003, 0.004)
**Model 2** ^a^				
Pack-years/1000	0.032**	0.010** ^†^	0.021**	0.008** ^†^
	0.042**	0.075**	0.0002**	0.0004**
	(0.038, 0.045)	(0.059, 0.091)	(0.0001, 0.0002)	(0.0003, 0.001)

WC: waist circumference

WHtR: waist circumference/height ratio

* p<0.05,

** p<0.01 for coefficients different from 0

^†^ P<0.05 for gender difference

^a^: Only nonsmokers and regular smokers were included.

Age, education, physical activity, alcohol use, survey site and BMI were included in models for adjustment.

**Fig 3:**
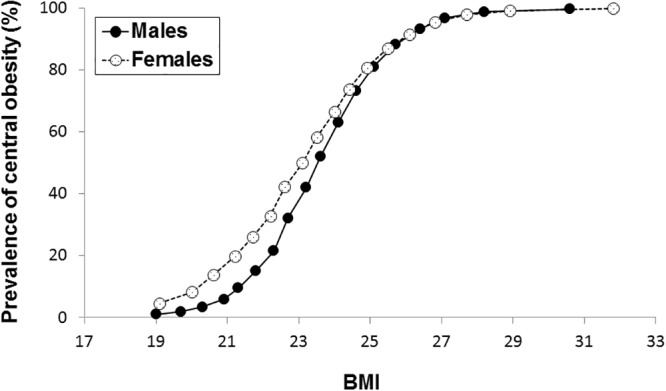
Prevalence of central obesity by 18 BMI subgroups in males and females.

Fig 3. illustrates the prevalence of central obesity by 18 BMI subgroups and gender. The prevalence of central obesity increased with increasing BMI at a faster rate at BMI levels below 24.0 in both males and females; the prevalence almost reached the ceiling in subgroups with a mean BMI above 28.0. Further, Pearson correlation coefficients between WHtR and BMI were 0.641, p<0.001 (males) and 0.550, p<0.001 (females) in the normal-weight group (BMI = 18.5–23.9); 0.487, p<0.001 (males) and 0.416, p<0.001 (females) in the over-weight group (BMI = 24.0–27.9); and 0.575, p<0.001 (males) and 0.542, p<0.001 (females) in the obesity group (BMI = 28.0–45.0). The positive BMI-WHtR correlations and negative smoking-BMI association indicated the importance of adjustment for BMI in the smoking-central obesity association analyses, especially in the normal-weight group.

In sensitivity analyses, the association between smoking and WHtR was examined in linear regression models by groups of normal-weight (BMI = 18.5–23.9), over-weight (BMI = 24.0–27.9) and obesity (BMI = 28.0–45.0), without and with adjusting for BMI along with other covariates. In males, the standardized linear regression coefficients of regular smoking for WC were -0.009* without adjusting for BMI and 0.040** with adjusting for BMI in the normal-weight group, 0.067** and 0.077** in the over-weight group and 0.066** and 0.072** in the obesity group (* p<0.05; ** p<0.01). In females, the standardized linear regression coefficients of regular smoking for WC were 0.001 without adjusting for BMI and 0.016** with adjusting for BMI in the normal-weight group, 0.016** and 0.018** in the over-weight group and 0.028** and 0.020** in the obesity group. In males, the standardized linear regression coefficients of regular smoking for WHtR were -0.032** without adjusting for BMI and 0.019** with adjusting for BMI in the normal-weight group, 0.043** and 0.054** in the over-weight group and 0.049** and 0.055** in the obesity group. In females, the standardized linear regression coefficients of regular smoking for WHtR were 0.001 without adjusting for BMI and 0.015** with adjusting for BMI in the normal-weight group, 0.013** and 0.015** in the over-weight group and 0.021** and 0.014** in the obesity group. The results of these sensitivity analyses indicated that the negative associations of regular smoking with WC and WHtR without adjusting for BMI were mainly because of the association patterns in the normal-weight group in males and females. The number of study subjects of the normal-weight group accounted for 54.2% of the entire study cohort.

## Discussion

Asian populations have a relatively lower BMI, but a greater prevalence of central obesity than Caucasian populations [5,7,11–16,28]. The CKB study, one of the largest cohort studies of chronic disease in the world, provides a great opportunity to investigate tobacco smoking and central obesity in Chinese population. In the current study, the prevalence of central obesity increased with increasing values of BMI, and female adults had a significantly higher prevalence of central obesity than male adults, especially in the normal-weight group. Of note, almost all Chinese adults (99.4%) with obesity (BMI = 28.0–45.0) in this cohort were centrally obese regardless of gender. Compared with males, females had a much lower prevalence of regular smokers (2.2% vs 60.6%), Regular smoking was associated with decreased BMI and increased WC and WHtR in both males and females when BMI was adjusted for in the model, and the association of smoking with WC, WHtR was much weaker in females than in males. These results indicate that female central obesity may have other important underlying mechanisms and risk factors in addition to tobacco smoking.

Epidemiologic studies have shown that body weight tends to be lower among smokers than among nonsmokers in many populations [22–24]. Smoking abstinence results in an increase in body weight for both males and females, and smoking is often considered as a way to control appetite and body weight [29]. The mechanisms that underlie this smoking-related weight phenomenon are complex and involve multiple neurochemical pathways. Most of the effects of smoking on body weight are mediated by nicotine inhaled from cigarette smoke. Nicotine acutely increases the levels of various neurotransmitters, such as the systemic release of catecholamines, dopamine and serotonin in the brain, suppresses appetite and consequently reduce food intake [23,30]. This process likely explains why smokers tend to decrease body weight, and why smoking cessation is frequently followed by weight gain [29]. As a result, the belief that cigarette smoking is helpful in controlling body weight has long been part of popular culture. Despite the universally accepted concept in this regard, there has been growing evidence that smoking is associated with abdominal fat accumulation; cross-sectional studies indicate that the prevalence of central obesity is higher in smokers than in nonsmokers [24,31–35]. Waist-to-hip ratio is positively associated with the number of pack-years of smoking [34], and there is a dose-response relation between waist-to-hip ratio and the number of cigarettes smoked [35]. The findings from the present study on the significantly inverse smoking-BMI association and positive smoking cessation-BMI association in both males and females are in concordance with observations from previous studies [22–24]. However, the association of regular smoking with WC and WHtR observed in the current study was dependent on the adjustment for BMI, especially in male normal-weight adults. After adjusting for BMI, regular smoking and the cumulative burden of smoking were significantly and positively associated with WC and WHtR; stratification analysis and model adjustment methods showed consistent results in this regard. The findings from the present study support the previous findings on the smoking-central obesity association [31–35]. Of interest, an important question has been raised in this study regarding whether BMI has to be adjusted for in the smoking-central obesity association analysis, particularly among normal-weight male adults.

By comparing the regression coefficients before and after adjustment for BMI in the model, completely opposite directions in the associations of regular smoking with WC and WHtR were noted in the current study. When BMI was not included in the model, current smoking showed a protective effect on WC and WHtR; whereas, current smoking was positively associated with these central obesity measures with BMI included in the model. The changes in the strength and direction of the association parameters suggested a considerable influence of BMI on these associations, particularly in the male normal-weight group as shown in the sensitivity analyses. When BMI was not adjusted for in regression analysis models, the negative association of regular smoking with WC (β = -0.009) and WHtR (β = -0.032) largely reflect the effect of smoking on BMI in the male normal-weight group. The smoking-BMI-central obesity relationship is similar to the mediation model proposed by Hernández-Díaz (Fig 3, panel 3.3) [36]. In the current analysis, the smoking→BMI indirect effect (-) and BMI↔WHtR correlation (+) are so strong that the absolute value of the negative total indirect effect (the product of the two indirect path effects) is supposed to be much greater than the positive value of smoking→WHtR direct effect (+). Thus, the total smoking→WHtR effect (the sum of indirect effect and direct effect) is expected to be negative without adjustment for BMI in the model as shown in Table 3. Apparently, whether or not BMI is adjusted for in the analysis model would lead to different conclusions in this regard. For this reason, the adjustment for BMI has been widely used in the risk factor-central obesity association analysis in the literature [37,38]. In addition, a traditional and powerful adjustment method, stratification analysis, was used to confirm this concept. The regression coefficients of regular smoking for WC and WHtR in the 18 BMI subgroups were all positive, which provided reliable evidence that regular smoking is positively associated with central obesity at all BMI levels. Results of the BMI stratification analyses support the argument that BMI needs to be included in the model for adjustment in the analyses related to smoking-central obesity association, especially for normal-weight adults. The phenomenon that the direction of any relation between two variables is reversed after a third variable is introduced is statistically known as “reversal paradox” [36,39]. The reversal paradox makes it very challenging to correctly interpret the findings seen in observational studies. Although BMI cannot be considered a confounder by definition in this scenario, the reversal paradox experienced in the current study points to the need for the adjustment for BMI in the smoking-central obesity association analysis.

The prevalence of overweight and obesity is higher among women than men, especially in developing countries [40]. Women often report consuming healthier foods, sex hormones (estrogens and testosterone), however, differentially affect adipocyte physiology and body fat distribution [41]. The increases in overweight and obesity in menopausal women are important public health concerns [42]. The prevalence of obesity increases significantly in American women after they reach age 40 [43]. In this study cohort, women showed a significantly higher prevalence of central obesity than men after age 45 in both total sample and the normal-weight group. On the other hand, the positive association between current smoking and central obesity measures was found to be significantly stronger in males than in females in this study. The higher, negative association between smoking and BMI in males (β = -0.093) than in females (β = -0.025), stronger correlation between WHtR and BMI (r = 0.641) in the male normal-weight group and higher prevalence of smokers in males may account for, in part, the gender-specific association between smoking and central obesity. The current observations suggest that female adults might have other more important mechanisms like sex hormones underlying central obesity in addition to tobacco smoking.

This population-based epidemiologic study has certain limitations. First, subjects voluntarily participated in the study, which might result in selection bias to some extent with respect to the population representativeness despite the large sample size. Second, the pathophysiological mechanisms underlying the association between smoking and central obesity cannot be investigated in the current study because the baseline survey data are observational in nature. Finally, the CKB study is not a nutritional survey, and detailed information on dietary patterns is not available. Therefore, dietary factors, which may be important, were not included in the current analyses.

In conclusion, the current study demonstrates that there are considerable differences in the prevalence of tobacco smokers and central obesity between male and female Chinese adults; smoking is significantly and positively associated with central obesity measured as WC and WHtR, with males having substantially stronger association parameters than females. Importantly, the association patterns of regular smoking with central obesity were largely dependent on BMI adjustment. The present study emphasizes the importance of adjustment for BMI in the smoking-central obesity association analyses, especially for normal-weight adults. Sophisticated statistical models like structural equation modeling or mediation analysis are needed to investigate this issue in depth. These gender-specific results indicate that other important underlying mechanisms like sex hormones may be involved in the development of central obesity in females in addition to tobacco smoking. The study findings underscore the importance of undertaking preventive strategies for central obesity through smoking behavior control.

## References

[1] Malik VS, Willett WC, Hu FB. Global obesity: trends, risk factors and policy implications. Nat Rev Endocrinol. 2013;9:13–27. 10.1038/nrendo.2012.199 23165161

[2] Flegal KM, Carroll MD, Kit BK, Ogden CL. Prevalence of obesity and trends in the distribution of body mass index among US adults, 1999–2010. JAMA. 2012;307:491–497. 10.1001/jama.2012.39 22253363

[3] Ogden CL, Carroll MD, Kit BK, Flegal KM. Prevalence of obesity and trends in body mass index among US children and adolescents, 1999–2010. JAMA. 2012;307:483–490. 10.1001/jama.2012.40 22253364 PMC6362452

[4] Wildman RP, Gu D, Muntner P, Wu X, Reynolds K, Duan X, et al Trends in overweight and obesity in Chinese adults: between 1991 and 1999–2000. Obesity (Silver Spring). 2008;16:1448–1453. 10.1038/oby.2008.208 18388899

[5] Xi B, Liang Y, He T, Reilly KH, Hu Y, Wang Q, et al Secular trends in the prevalence of general and abdominal obesity among Chinese adults, 1993–2009. Obes Rev. 2012;13:287–296. 10.1111/j.1467-789X.2011.00944.x 22034908 PMC3276709

[6] Flegal KM, Kit BK, Orpana H, Graubard BI. Association of all-cause mortality with overweight and obesity using standard body mass index categories: a systematic review and meta-analysis. JAMA. 2013;309:71–82. 10.1001/jama.2012.113905 23280227 PMC4855514

[7] Zhou B-F. Predictive values of body mass index and waist circumference for risk factors of certain related diseases in Chinese adults—study on optimal cut-off points of body mass index and waist circumference in Chinese adults. Biomed Environ Sci. 2002;15:83–96. 12046553

[8] Whitlock G, Lewington S, Sherliker P, Clarke R, Emberson J, Halsey J, et al Body-mass index and cause-specific mortality in 900 000 adults: collaborative analyses of 57 prospective studies. Lancet. 2009;373:1083–1096. 10.1016/S0140-6736(09)60318-4 19299006 PMC2662372

[9] Cameron AJ, Boyko EJ, Sicree RA, Zimmet PZ, Söderberg S, Alberti KG, et al Central obesity as a precursor to the metabolic syndrome in the AusDiab study and Mauritius. Obesity (Silver Spring). 2008;16:2707–2716. 10.1038/oby.2008.412 18820650

[10] Srinivasan SR, Wang R, Chen W, Wei CY, Xu J, Berenson GS. Utility of waist-to-height ratio in detecting central obesity and related adverse cardiovascular risk profile among normal weight younger adults (from the Bogalusa Heart Study). Am J Cardiol. 2009;104:721–724. 10.1016/j.amjcard.2009.04.037 19699351

[11] Hsieh SD, Muto T. Metabolic syndrome in Japanese men and women with special reference to the anthropometric criteria for the assessment of obesity: Proposal to use the waist-to-height ratio. Prev Med (Baltim). 2006;42:135–139. 16376978 10.1016/j.ypmed.2005.08.007

[12] Hsieh SD, Muto T. The superiority of waist-to-height ratio as an anthropometric index to evaluate clustering of coronary risk factors among non-obese men and women. Prev Med (Baltim). 2005;40:216–220. 10.1016/j.ypmed.2004.05.025 15533532

[13] Deurenberg P, Deurenberg-Yap M, Guricci S. Asians are different from Caucasians and from each other in their body mass index/body fat per cent relationship. Obes Rev. 2002;3:141–146. 12164465 10.1046/j.1467-789x.2002.00065.x

[14] Low S, Chin MC, Ma S, Heng D, Deurenberg-Yap M. Rationale for redefining obesity in Asians. Ann Acad Med Singapore. 2009;38:66–69. 19221673

[15] WHO Expert Consultation. Appropriate body-mass index for Asian populations and its implications for policy and intervention strategies. Lancet. 2004;363:157–163. 14726171 10.1016/S0140-6736(03)15268-3

[16] Ramachandran A, Chamukuttan S, Shetty SA, Arun N, Susairaj P. Obesity in Asia—is it different from rest of the world. Diabetes Metab Res Rev. 2012;28 Suppl 2:47–51. 10.1002/dmrr.2353 23280866

[17] Cheng TO. The current state of cardiology in China. Int J Cardiol. 2004;96:425–439. 15301897 10.1016/j.ijcard.2003.10.011 PMC7127163

[18] Reynolds K, Gu D, Whelton PK, Wu X, Duan X, Mo J, et al Prevalence and risk factors of overweight and obesity in China. Obesity (Silver Spring). 2007;15:10–18. 17228026 10.1038/oby.2007.527

[19] Björntorp P. Visceral obesity: a “civilization syndrome”. Obes Res. 1993;1:206–222. 16350574 10.1002/j.1550-8528.1993.tb00614.x

[20] Wadden TA, Webb VL, Moran CH, Bailer BA. Lifestyle modification for obesity: new developments in diet, physical activity, and behavior therapy. Circulation. 2012;125:1157–1170. 10.1161/CIRCULATIONAHA.111.039453 22392863 PMC3313649

[21] Hebden L, Chey T, Allman-Farinelli M. Lifestyle intervention for preventing weight gain in young adults: a systematic review and meta-analysis of RCTs. Obes Rev. 2012;13:692–710. 10.1111/j.1467-789X.2012.00990.x 22413804

[22] Molarius A, Seidell JC, Kuulasmaa K, Dobson AJ, Sans S. Smoking and relative body weight: an international perspective from the WHO MONICA Project. J Epidemiol Community Health. 1997;51:252–260. 9229053 10.1136/jech.51.3.252 PMC1060469

[23] Audrain-McGovern J, Benowitz NL. Cigarette smoking, nicotine, and body weight. Clin Pharmacol Ther. 2011;90:164–168. 10.1038/clpt.2011.105 21633341 PMC3195407

[24] Chiolero A, Faeh D, Paccaud F, Cornuz J. Consequences of smoking for body weight, body fat distribution, and insulin resistance. Am J Clin Nutr. 2008;87:801–809. 18400700 10.1093/ajcn/87.4.801

[25] Li L, Lv J, Guo Y, Collins R, Chen JS, Peto R, et al The China Kadoorie Biobank: related methodology and baseline characteristics of the participants. Zhonghua Liu Xing Bing Xue Za Zhi. 2012;33:249–255. 22613372

[26] Chen Z, Chen J, Collins R, Guo Y, Peto R, Wu F, et al China Kadoorie Biobank of 0.5 million people: survey methods, baseline characteristics and long-term follow-up. Int J Epidemiol. 2011;40:1652–1666. 10.1093/ije/dyr120 22158673 PMC3235021

[27] Du H, Bennett D, Li L, Whitlock G, Guo Y, Collins R, et al Physical activity and sedentary leisure time and their associations with BMI, waist circumference, and percentage body fat in 0.5 million adults: the China Kadoorie Biobank study. Am J Clin Nutr. 2013;97:487–496. 10.3945/ajcn.112.046854 23364014 PMC4345799

[28] Misra A, Shrivastava U. Obesity and dyslipidemia in South Asians. Nutrients. 2013;5:2708–2733. 10.3390/nu5072708 23863826 PMC3738996

[29] Williamson DF, Madans J, Anda RF, Kleinman JC, Giovino GA, Byers T. Smoking cessation and severity of weight gain in a national cohort. N Engl J Med. 1991;324:739–745. 1997840 10.1056/NEJM199103143241106

[30] Benowitz NL. Nicotine addiction. N Engl J Med. 2010;362:2295–2303. 10.1056/NEJMra0809890 20554984 PMC2928221

[31] Wakabayashi I. Relationship between smoking and metabolic syndrome in men with diabetes mellitus. Metab Syndr Relat Disord. 2014;12:70–78. 10.1089/met.2013.0110 24266721

[32] Kim JH, Shim KW, Yoon YS, Lee SY, Kim SS, Oh SW. Cigarette smoking increases abdominal and visceral obesity but not overall fatness: an observational study. PLoS One. 2012;7:e45815 10.1371/journal.pone.0045815 23029258 PMC3454366

[33] Shimokata H, Muller DC, Andres R. Studies in the distribution of body fat. III. Effects of cigarette smoking. JAMA. 1989;261:1169–1173. 2915440

[34] Canoy D, Wareham N, Luben R, Welch A, Bingham S, Day N, et al Cigarette smoking and fat distribution in 21,828 British men and women: a population-based study. Obes Res. 2005;13:1466–1475. 16129730 10.1038/oby.2005.177

[35] Jee SH, Lee SY, Nam CM, Kim SY, Kim MT. Effect of smoking on the paradox of high waist-to-hip ratio and low body mass index. Obes Res. 2002;10:891–895. 12226137 10.1038/oby.2002.122

[36] Hernández-Díaz S, Schisterman EF, Hernán MA. The birth weight "paradox" uncovered? Am J Epidemiol. 2006;164:1115–1120. 16931543 10.1093/aje/kwj275

[37] Heid IM, Jackson AU, Randall JC, Winkler TW, Qi L, Steinthorsdottir V, et al Meta-analysis identifies 13 new loci associated with waist-hip ratio and reveals sexual dimorphism in the genetic basis of fat distribution. Nat Genet. 2010;42:949–960. 10.1038/ng.685 20935629 PMC3000924

[38] Liu CT, Monda KL, Taylor KC, Lange L, Demerath EW, Palmas W, et al Genome-wide association of body fat distribution in African ancestry populations suggests new loci. PLoS Genet. 2013;9(8):e1003681 10.1371/journal.pgen.1003681 23966867 PMC3744443

[39] Tu Y-K, West R, Ellison GTH, Gilthorpe MS. Why evidence for the fetal origins of adult disease might be a statistical artifact: the “reversal paradox” for the relation between birth weight and blood pressure in later life. Am J Epidemiol. 2005;161:27–32. 15615910 10.1093/aje/kwi002

[40] Kanter R, Caballero B. Global gender disparities in obesity: a review. Adv Nutr 2012;3:491–498. 10.3945/an.112.002063 22797984 PMC3649717

[41] Lizcano F, Guzmán G. Estrogen Deficiency and the Origin of Obesity during Menopause. Biomed Res Int. 2014;2014:757461 10.1155/2014/757461 24734243 PMC3964739

[42] Wietlisbach V, Marques-Vidal P, Kuulasmaa K, Karvanen J, Paccaud F. The relation of body mass index and abdominal adiposity with dyslipidemia in 27 general populations of the WHO MONICA Project. Nutrition, Metabolism and Cardiovascular Diseases. 2013;2:432–442. 10.1016/j.numecd.2011.09.002 22209742

[43] Flegal KM, Carroll MD, Ogden CL, Curtin LR. Prevalence and trends in obesity among US adults, 1999–2008. JAMA. 2010;303:235–241. 10.1001/jama.2009.2014 20071471

